# Intrauterine growth restriction - impact on cardiovascular diseases later in life

**DOI:** 10.1186/s40348-018-0082-5

**Published:** 2018-03-20

**Authors:** Carlos Menendez-Castro, Wolfgang Rascher, Andrea Hartner

**Affiliations:** Department of Pediatrics and Adolescent Medicine, University of Erlangen-Nuremberg, Loschgestrasse 15, 91054 Erlangen, Germany

**Keywords:** Intrauterine growth restriction, Fetal programming, Cardiovascular disease

## Abstract

Intrauterine growth restriction (IUGR) is a fetal pathology which leads to increased risk for certain neonatal complications. Furthermore, clinical and experimental studies revealed that IUGR is associated with a significantly higher incidence of metabolic, renal and cardiovascular diseases (CVD) later in life. One hypothesis for the higher risk of CVD after IUGR postulates that IUGR induces metabolic alterations that then lead to CVD.

This minireview focuses on recent studies which demonstrate that IUGR is followed by early primary cardiovascular alterations which may directly progress to CVD later in life.

## Background

Intrauterine growth restriction (IUGR) affects 3–7% of all newborns. As a consequence of maternal, placental or fetal pathology the fetus cannot fully exploit its growth potential [1]. While the term “small for gestational age” (SGA) describes a newborn with a birth weight less than the 10th percentile, IUGR requires a pathological retardation of intrauterine growth velocity clearly highlighted by a characteristic kink in the curve of intrauterine growth [2].

Newborns with IUGR exhibit significantly increased morbidity immediately after birth (e.g. hypoglycaemia, hypothermia, infant respiratory distress syndrome). Later in life former IUGR-patients were found to have a significantly higher incidence of renal, cardiovascular and metabolic diseases, the same ailments that are also the most frequent causes of morbidity and mortality in the western world [3–5].

The underlying mechanisms leading from fetal undersupply to the development of diseases in adulthood are not fully understood. In this context Barker et al. hypothesize that intrauterine undernutrition compromises growth and differentiation of organs during the vulnerable phase of fetal development that results in persistent alterations of the organism and leads to the development of secondary diseases later in life [6–8].

The shortage of nutrients during fetal development observed in IUGR is commonly replaced by an adequate nutrient supply after birth. The hypothesis of the “thrifty phenotype” considers the mismatch between intrauterine and postnatal supply with nutrients in IUGR individuals to be the underlying cause of secondary pathologies. In a fetus adapted to shortage of nutrients during intrauterine development postnatal hyperalimentation leads initially to excessive catch up growth and later to metabolic, cardiovascular and renal diseases [9].

As IUGR is associated with a higher incidence of metabolic syndrome, it appears conceivable that cardiovascular dysfunction observed after IUGR is secondary to diabetes, dyslipidemia or hypertension [10]. However, growing evidence suggests that IUGR directly causes cardiovascular alterations independent of pre-existing metabolic disease. Recent clinical and animal studies identified candidate mechanisms that may mediate the development of cardiovascular alterations in the setting of IUGR consistent with the hypothesis of perinatal programming [11, 12].

### Myocardial function and structure after IUGR

In the heart, a number of studies revealed myocardial alterations induced by IUGR, that can be detected long before the onset of metabolic disease and arterial hypertension. IUGR affected cardiac development and significantly reduced the number of cardiomyocytes at the time of birth [13, 14]. Studies in humans revealed early and persistent alterations of myocardial structure and decreased cardiac function detected by echocardiography in fetal, neonatal and juvenile patients with IUGR [15–17]. These results were supported by findings in an animal model of IUGR exhibiting echocardiographic signs of cardiac dysfunction accompanied by a more distensible myocardium in the absence of arterial hypertension [18]. As a possible underlying mechanism, changes in the expression pattern of Titin (Ttn) after IUGR was observed: The passive elasticity of cardiomyocytes is modulated by alternative splicing of titin, a structural protein of the myocardial sarcomere, resulting in the two isoforms N2BA and N2B. Corresponding to the echocardiographic signs of a more distensible myocardium, relative overexpression of the less rigid isoform N2BA was observed in IUGR animals [18].

**Fig. 1:**
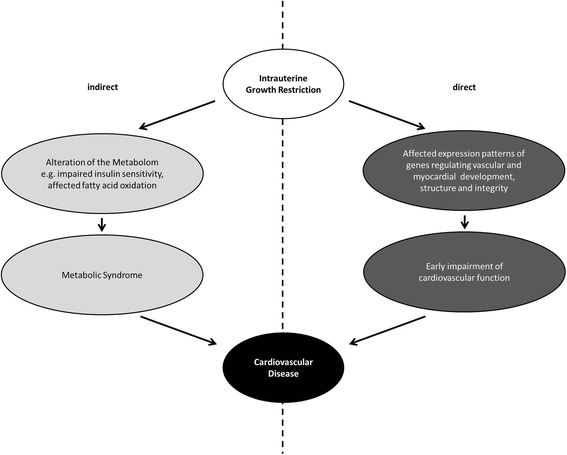
Direct and indirect pathways leading to cardiovascular disease in IUGR

Moreover, assessment of inflammatory and profibrotic markers revealed an early induction of the expression of transforming growth factor beta (TGF-ß), connective tissue growth factor (CTGF) and microfibrillar matrix molecules in the myocardium of juvenile IUGR animals without evidence of metabolic syndrome [19]. This supports the notion of early and direct molecular changes in the cardiovascular system (Fig. 1).

### Atherosclerosis, vascular remodeling and fibrosis after IUGR

IUGR-induced metabolic disease (including dyslipidemia and insulin resistance) may likewise indirectly lead to atherosclerosis [10]. In addition, several studies provided evidence that IUGR is accompanied by early structural alterations in blood vessels [20–23]. Vascular remodeling favors the development of atherosclerosis [24]. In an animal model of vascular remodeling induced by unilateral ligation of the A. carotis communis significantly increased neointima formation and media thickness was observed in juvenile IUGR rats in the absence of metabolic disease [25]. Moreover, dedifferentiation of vascular smooth muscle cells (VSMC) was more prominent and collagen deposition in the media was increased after IUGR [25].

But even in the absence of experimental vascular disease, primary vascular alterations were detected in normotensive IUGR animals: A significantly increased expression of connective tissue growth factor (CTGF) was observed in aortas of neonatal animals after IUGR. Collagen I and collagen IV deposition in the aorta was more prominent in juvenile IUGR animals [19]. Recent studies examined IUGR-induced molecular mechanisms of endothelial dysfunction that favor the development of atherosclerosis. Oliveira et al. detected lower NO levels and increased eNOS phosphorylation in thoracic aortas of IUGR rats as a sign of endothelial dysfunction [26]. Taken together, these observations indicate that IUGR renders individuals more susceptible to the development of atherosclerotic lesions and vascular dysfunction and thus to cardiovascular-related diseases later in life.

## Conclusions

IUGR favors the development of secondary cardiovascular diseases later in life which are among the most frequent causes of morbidity and mortality and constitute a significant proportion of healthcare-related expenditures in the western world. Cardiovascular disease appears not only to be a consequence of metabolic syndrome, but also caused by direct effects on cardiac and vasculature structure and function in individuals with IUGR. A more detailed knowledge of underlying disease mechanisms is likely to advance prevention and treatment of IUGR and its complications, and thereby improve long-term outcomes for patients with IUGR.

## Abbreviations

CTGF: connective tissue growth factor
eNOS: endothelial NO-Synthase
IUGR: intrauterine growth restriction
NO: nitric oxide
SGA: small for gestational age
TGF-ß: transforming growth factor beta
VSMC: vascular smooth muscle cells
